# Development of Personas to Communicate Narrative-Based Information About the HPV Vaccine on Twitter

**DOI:** 10.3389/fdgth.2021.682639

**Published:** 2021-08-04

**Authors:** Philip M. Massey, Shawn C. Chiang, Meredith Rose, Regan M. Murray, Madeline Rockett, Elikem Togo, Ann C. Klassen, Jennifer A. Manganello, Amy E. Leader

**Affiliations:** ^1^Department of Community Health and Prevention, Dornsife School of Public Health, Drexel University, Philadelphia, PA, United States; ^2^Department of Health Policy, Management and Behavior, School of Public Health, University at Albany, State University of New York (SUNY), Albany, NY, United States; ^3^Division of Population Science, Department of Medical Oncology, Thomas Jefferson University, Philadelphia, PA, United States

**Keywords:** personas, HPV vaccination, vaccine hesitancy, Twitter, narrative communication, health communication

## Abstract

**Introduction:** Personas are based on real-life typologies of people that can be used to create characters and messages to communicate important health information through relatable narrative storylines. Persona development is data-driven and can involve multiple phases of formative research and evaluation; however, personas are largely underutilized in digital health research. The purpose of this study was to create and document persona development to deliver narrative-focused health education for parents on Twitter with the goal of increasing uptake of HPV vaccination among adolescents.

**Methods:** Leveraging data from a mixed-method study conducted in the U.S. with a diverse population of parents with adolescents ages 9–14, we used both qualitative and quantitative data (e.g., the National Immunization Survey—Teen, focus groups, and social media) to create personas. These data sources were used to identify and develop key characteristics for personas to reflect a range of parents and their diverse understandings and experiences related to HPV vaccination. A parent advisory board provided insight and helped refine persona development.

**Results:** Four personas emerged and were characterized as the (1) Informed Altruist, (2) Real Talker, (3) Information Gatherer, and (4) Supporter. Characteristics differed across personas and provided insights into targeted narrative strategies. Described attributes included demographics, psychographics, communication style, vaccine goals and aspirations, vaccine challenges and frustrations, and vaccine hesitancy.

**Discussion:** This work demonstrates how multiple data sources can be used to create personas to deliver social media messages that can address the diverse preferences and needs of parents for HPV vaccine information. With increasing usage of social media for health information among parents, it is important for researchers to consider marketing and design thinking to create health communication messages that resonate with audiences.

## Introduction

From the early use of parables and fables, communicators across history have used stories to share ideas with diverse audiences. Storytelling has been established as a way to engage audiences, present important ideas through relatable scenarios and characters, and create and make use of memorable and persuasive messages through story plots and outcomes (1). In health education, the use of stories continues to evolve but remains an important strategy in persuasive communication, especially for audiences of diverse backgrounds and literacy levels (2). Entertainment education, or EE, is a specific area of health communication that leverages songs, televised stories (telenovelas), social media (including games and blogs) and other mediated communication to combine theory-based behavior change with storytelling (3).

Storytelling has been used for health education in various environments including broadcast television (4, 5), digital spaces (6, 7), and in-person healthcare delivery (8). The storytelling approach has the potential to reach and resonate with different populations and communities with diverse backgrounds and lived experiences (9–12), and can be a useful strategy to communicate science to non-expert audiences (13). This may be particularly relevant to vaccine science and communication, as storytelling has the potential to address concerns that are rooted more in emotions than lack of evidence (14, 15). Specific to the human papillomavirus (HPV) vaccine, storytelling has fostered vaccine confidence when shared through multimedia (16) and interpersonal conversations (17). Indeed, a previous study found that peer-expert narrative intervention nearly doubled the HPV vaccination initiation rate compared to a non-narrative approach (18). Additionally, HPV vaccine narratives or stories, as compared to pure informational resources, have been shown to garner more engagement on social media (19). While the use of stories and sharing of experiences on social media may vary by user type (i.e., parents, teens, health providers) (20), various aspects of storytelling have become a commonly seen characteristic of social media posts.

The use of characters and scenarios in storytelling and persuasive communication can influence many elements which are known to be fundamental drivers of behavior and behavior change, including audience beliefs about norms (what people like me typically do or should do), values (what are the typical costs and benefits of a hypothetical action in my community), problem-solving strategies, and interpersonal interactions between partners, families and communities (21). Characters in health communication stories must be relatable enough to engage audience interest. At the same time they need to be credible in their appearance and life situation, attitudes and beliefs, and actions and consequences (22). They should also elicit an emotional connection for audience members, so that their experiences are felt in a meaningful way.

Personas can be used to inform character development, and can be powerful tools through which to communicate narratives and storytelling for health education, training, and research. Personas are hypothetical archetypal representations of actual target users with details such as demographic information, behaviors, goals, professions, etc., which are intended to represent a user and can be used to communicate key motivations, concerns, and interests (23). Often used in human-centered design (HCD) approaches, a problem-focused framework that emerged from the fields of industrial design (24), personas are increasingly applied in health-related solutions (25–27). The HCD process typically involves interviews, observation, and immersion in a user's context to develop user personas and use scenarios (23). Health studies have explored topics such as the use of personas to improve communicable disease workflow in public health (28), the ways personas provide information in online communities (29), and how people create their own online personas (30). Yet, we found little evidence of research examining the development of personas to utilize for disseminating health information through social media.

Bridging together the themes of digital health communication, storytelling, and design thinking, personas can provide a unique mechanism to create and communicate credible, accurate, and timely information in a meaningful and memorable way. Much health information is provided as information and facts designed to provide evidence to someone making health-related decisions, but as health interventions continue to emerge on social media and other online platforms, researchers and practitioners may seek to develop personas to deliver messages and materials. However, little has been documented on describing this approach for health messages on social media and how it can be informed by formative research and evaluation. To address this gap in knowledge, we provide a case-based and evidence-informed example of this approach, describing how personas can be developed to use on social media for targeting parents' decisions to vaccinate their children against the human papillomavirus (HPV). Specifically, the purpose of this project was to develop personas, the characters and their backgrounds, that can be used to communicate about the HPV vaccine and foster confidence among parents on Twitter.

## Methods

**Figure 1:**
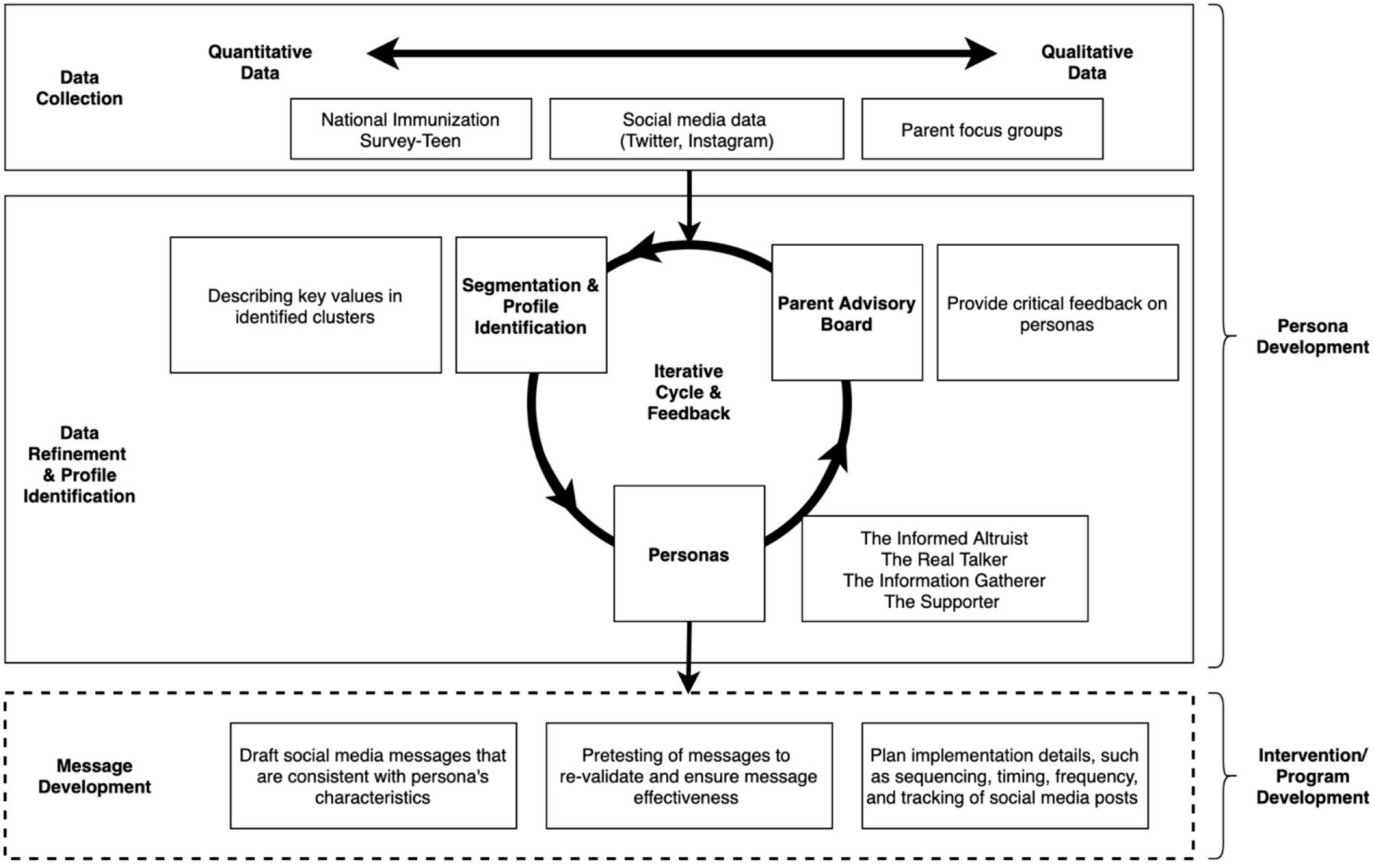
Process used for persona development, including data collection, data refinement, and profile identification phases.

We utilized mixed methods to develop personas that represented both a breadth and depth of HPV vaccine understanding and experiences among parents and our approach was informed by a number of studies (31–33) that had used human-centered design. To interpret data and refine persona development, we created and worked with a parent advisory board (PAB). For persona presentation, we created persona profiles that highlight demographics, psychographics (34) (i.e., psychological traits, such as personality, values, desires, and lifestyle), communication style, vaccine goals and aspirations, and vaccine challenges and frustrations. Figure 1 displays the process used for persona development. In brief, we conceptualized our process in three stages, (1) Data Collection, (2) Data Refinement and Profile Identification, and (3) Message Development. The current manuscript focuses on the first two stages.

### Data Collection

To collect qualitative data, in May 2020 we conducted virtual focus groups (*n* = 6) with parents (*n* = 48) from across the U.S. to gain a deeper understanding of what they considered and how they came to the decision to get their child vaccinated against HPV (35). We focused on their experiences and conversations (or lack thereof) with other parents about the HPV vaccine, and if hearing from other parents about their experiences was important to them when deciding to vaccinate. Parents were recruited from a national panel managed by Ipsos, a market research firm. To be eligible to participate in focus groups, parents had child(ren) ages 9–14, used Twitter at least once a week, did not hold anti-vaccine views, and spoke English. Participants were considered holding anti-vaccine views if they scored <2 average (on a Likert scale 1–4) where 1 is “strongly disagree” and 4 is “strongly agree,” on three HPV attitude questions—“How effective do you think the HPV vaccine is in preventing cancers caused by HPV?,” “How much do you agree or disagree that the HPV vaccine is an important part of your child's health?,” and “How much do you agree or disagree that the HPV vaccine is safe?.” Using a text-based focus group discussion format, we discussed their experiences getting information about the HPV vaccine and using Twitter to learn about health topics more generally. Four members of the research team used NVivo 12 to conduct qualitative analysis for the focus groups and structured findings by themes including content, delivery, and source of information. We utilized both a deductive approach with a priori codes based on the focus group guide as well as an inductive approach that allowed additional themes to emerge that were relevant to our research questions. Cohen's kappa was calculated for each coder pair, and then averaged. An average inter-rater reliability of 0.65 was achieved overall and any subsequent differences were reviewed and reconciled in line with the codebook. A full description of procedures and findings from the focus group study are published providing further detail (35).

For quantitative data, we examined the 2018 National Immunization Survey (NIS)-Teen (36) to identify parental vaccine concerns at a population-level (37). Parents who answered “not too likely,” “not likely at all,” or “not sure/do not know” to “How likely is it the teen will receive HPV vaccination in the next 12 months?” were considered to be HPV vaccine hesitant. The NIS-Teen provides data on common concerns described by parents when considering the HPV vaccine for their adolescent by asking “What is the MAIN reason [teen name] will not receive any HPV shots in the next 12 months?” and response options included vaccine effectiveness concerns, vaccine safety concerns, lack of physician recommendation, adolescent not sexually active, among others. Chi-squared tests were used to compare differences in gender of parent, as well as levels of hesitancy to vaccinate adolescent and reasons for delay. Analyses were conducted in SAS 9.4 (Cary, NC) using survey weighting methods described in NIS-Teen data user guide (36).

Finally, we utilized both quantitative and qualitative observational data from social media, specifically Twitter (38) and Instagram (39), to characterize social media-level communication patterns. The study team used an application programming interface (API) to collect public posts from these platforms that included relevant hashtags such as #hpvvaccine. We content analyzed social media posts and also examined post metadata, including likes and shares. Bivariate analyses allowed us to examine whether the content of the post was associated with engagement (e.g., comparing the average number of likes for information-focused vs. narrative-focused posts). In addition, through a network analysis of anti-HPV vaccine Instagram posts, we characterized how misinformation specific to the HPV vaccine was described and communicated (19).

### Persona Development

To begin integrating data and developing our personas, we categorized the combined data (focus groups, NIS-Teen, and social media) by vaccine attitudes (i.e., supportive, hesitant), vaccine influences (i.e., individual, societal), and vaccine-specific issues (i.e., awareness, safety). We identified emerging patterns in characteristics and used these to develop our initial four clusters of potential personas. Of note, we focused on supportive and hesitant vaccine beliefs for our persona development, and excluded anti-vaccine beliefs, as our goal was to create personas to deliver information to parents who are considering or are unsure of the HPV vaccine for their child. From this point, we introduced psychographics (i.e., attitudes, aspirations, motivations) to our data-derived clusters, and merged and shifted characteristics from different personas based on iterative evaluation of the combined datasets and evolving themes to arrive at our first iteration of the four personas.

### Data/Persona Refinement

We assembled a diverse parent advisory board (PAB) by recruiting on Twitter, ultimately choosing six parents (five female, one male) who identified as African-American, South Asian-American, and Caucasian, from across six states in the U.S., including urban and suburban, with children ages 11–12. Vaccinating their adolescent against HPV was not required, such that some parents on the PAB had gotten their children the HPV vaccine, while others were still deciding; however, none of the parents had decided against vaccinating their adolescent. The PAB provided expert insights and feedback on persona characteristics, lifestyle, vaccine motivations, vaccine issues, communication, contextual influences, and individual influences. Over a 6-month period, we held monthly meetings with the PAB to discuss and refine the personas.

Before each meeting (*n* = 6), we asked that all PAB members complete a workbook with persona development materials and targeted questions to gather extensive feedback. The questions aimed to capture thoughts about persona descriptions while connecting their lived experiences to the personas. We have provided two sample workbooks as Supplementary Materials. Once the workbooks were completed, the study team compiled summary documents and conducted a thematic analysis of all responses, identifying recurring themes within each section of each workbook.

We used the widely accepted 3C model of vaccine hesitancy (40) to identify distinct vaccine beliefs for each of the personas related to confidence, complacency, and convenience. We ranked each persona on a Likert scale (low, somewhat, moderate, and high) on the three metrics. For confidence and convenience, a high score correlated with positive associations with the HPV vaccine, while a low score correlated with more negative associations with the HPV vaccine. Confidence refers to trust in the effectiveness and safety of vaccines, the system that delivers them—including the reliability of the health professional—and/or the motivations of policymakers who make determinations about vaccines. Convenience refers to the degree to which the comfort, time, place, and quality of a vaccine affects uptake of the vaccine. Complacency was reversed scored, meaning that a high score correlated with more negative associations and a low score correlated with more positive associations. Complacency refers to a low perceived risk of vaccine-preventable diseases and therefore it is assumed vaccines are not needed.

### Persona Profile Identification

The final four personas were described to highlight their unique characteristics, including demographics, goals and aspirations, challenges and frustrations, communication needs, as well as confidence, convenience, and complacency of the 3C model. Background information included demographics such as gender, age, marital status, education level, and child(ren) status, as well as lifestyle information such as occupation, extracurriculars, and personality characteristics. Goals and aspirations correlated to each persona's attitudes and beliefs in regards to promoting health behaviors related to HPV vaccine uptake. Challenges and frustrations correlated to the obstacles each persona faced in terms of processing health information, engaging with others, and impediments to action regarding the HPV vaccine. Communication needs described the communication style, preferences, and social media activity/engagement utilized by the personas to connect with others in their social networks both on and offline.

## Results

Table 1 provides a joint display that describes how findings from each data source (i.e., focus groups, NIS-Teen, social media, and PAB) were integrated and used to inform the development of each persona. This method of organization was used to demonstrate how deliberate we were in using the data to inform the development of specific personas. Cross-cutting themes drawn from the key findings across different data sources emerged to ground our persona development. Furthermore, integrating findings from across multiple data sources both complemented and expanded the understanding of each persona, particularly as it related to HPV vaccine beliefs, motivations, and behaviors.

**Table 1:** Data type and content used to inform development of each persona.

**Persona**	**Data source**			
	**Social media**	NIS-Teen^*^	**Focus groups**	**Advisory board**
Informed Altruist	Informational resources an important foundation and most prevalent.	Likely to say “yes” to the vaccine. Few safety or effectiveness concerns	Parents want evidence to help them support their decision to vaccinate.	Has a powerful job and is non-confrontational when it comes to vaccine conversations
Real Talker	Myth busting and addressing conspiracy theories and lies.	Confident speaking to parents who are “not likely at all” to vaccinate adolescent and have high safety concerns	In regards to vaccine experiences, parents thought positive experiences should be highlighted and made more memorable.	Young parent who draws from personal experiences
Information Gatherer	Misinformation is prevalent and at times difficult to decipher—addressing distortive tactics.	Need strong provider recommendation and credible, accurate information	Parents want to hear from other parent experiences but also wanted to have data to support these narratives and stories.	Wants to make best choice for family. Afraid of making the wrong decision
Supporter	Personal stories received the most engagement.	More likely to be a father-figure who may not have high HPV vaccine awareness or knowledge	Talking to other parents about the HPV vaccine or vaccines in general is a difficult. Many parents are unsure how/where these interactions take place.	Seen as a mentor and active listener

* *NIS-Teen, National Immunization Survey—Teen*.

In *focus groups*, parents stated that they wanted to hear about experiences from other parents but did not know how to start this conversation. Parents also indicated that while positive experiences with the HPV vaccine were far more common, the negative experiences were often more memorable. Additionally, parents thought that hearing about other parent experiences would be an important complement to data and resources. That is, experiences alone would not be sufficient to help strengthen their confidence, but were a necessary complement to the science and evidence supporting the HPV vaccine.

*NIS-Teen data* revealed that among parents whose adolescents had not started or started but not completed the HPV vaccination in 2018, 30% were not likely at all, 15% were not too likely, and 9% were unsure about vaccinating their adolescents in the next 12 months. Compared to “not too likely” parents, “not likely at all” parents had greater concerns for HPV vaccine safety (14 vs. 25%), fewer received a physician recommendation (17 vs. 8%), and fewer lacked knowledge about the vaccine (11 vs. 3%) (all *p* < 0.0001). Mothers, compared to fathers, were more likely to have concerns about safety and side effects (21 vs. 11%). However, fathers were more likely to report not knowing about HPV vaccine (10 vs. 7%) and fewer received a physician recommendation (17 vs. 12%) (all *p* < 0.0001). These data indicated that current HPV vaccine attitudes and knowledge vary depending on specific subgroups and tailored communication should be considered.

*Social media data* demonstrated that positive sentiment about the HPV vaccine was more prevalent than negative, and most often communication through informational posts. Specific to Twitter data (*n* = 193,379 tweets), the majority of content was positive and content shared by parents was more often personal experiences compared with information or resources. On Instagram (*n* = 508 posts), while more posts communicated positive sentiment about the HPV vaccine, posts with negative sentiment were more often about personal stories and experiences, and also received more engagement (i.e., more likes). Social media data also revealed that misinformation takes many forms, but at the same time demonstrates patterns and consistent elements. For instance, conspiracy theories and recently uncovered “unknown facts” about the HPV vaccine clustered together, most often with the sentiment of purporting to reveal a lie (19). Vaccine injury stories were told through a distortive lens often implying correlation or comparison between two unrelated pieces of information or evidence.

*Parents on the advisory board* provided critical data points to inform the development of personas. The two sample workbooks provided as Supplementary Materials were used to gather individual data from PAB members, and we followed this with a group discussion on the workbook topics. Personas evolved based on this feedback, as seen when comparing the persona names across the two workbooks—for example, the Researcher became the Information Gather.

The PAB described how they personally relate to each persona, which added a final layer of realism to the persona descriptions. The PAB either saw themselves or others they knew in the personas.

- “*Many elements of this description remind me of myself or some in my closest circle.”*
- “*As I was reading I could name specific people in my life who fit the characteristics of each character.”*

They proposed demographics, communication styles, strengths, and challenges for each of the personas based on lived experiences.

- “*As a woman, I think I do think of this person [Informed Altruist] as a college educated woman who may or may not have left the work force and now has the time and means to partake in volunteer positions. I think this person might also work for a non-profit.”*
- “*They [the Real Talker] are very articulate at getting their point across and with ease. They can be very demanding at times.”*

The PAB suggested that it would be important to highlight specific fears around vaccines (e.g., making the wrong decision or not feeling confident in a choice).

- “*She [information gatherer] is well-intentioned and warm-hearted, but flighty. She would rather not make a decision if she thinks she may make a wrong decision.”*
- “*I wonder if instead of the [information gatherer] being scared to make the wrong choice it is more wanted to make the best choice for their family.”*

The advisory board also discussed how they personally communicate with other parents around vaccines, and how we could incorporate those specific details into the personas (i.e., confrontational vs. non-confrontational on social media, being an active listener).

- “*I would never want to suggest to another parent that he or she is not protecting their child if they do their research and then make a choice not to vaccinate.”*
- “*I think many of us in this category are averse to conflict, which also compounds the stress of being challenged if our facts aren't airtight.”*

### Personas

**Figure 2:**
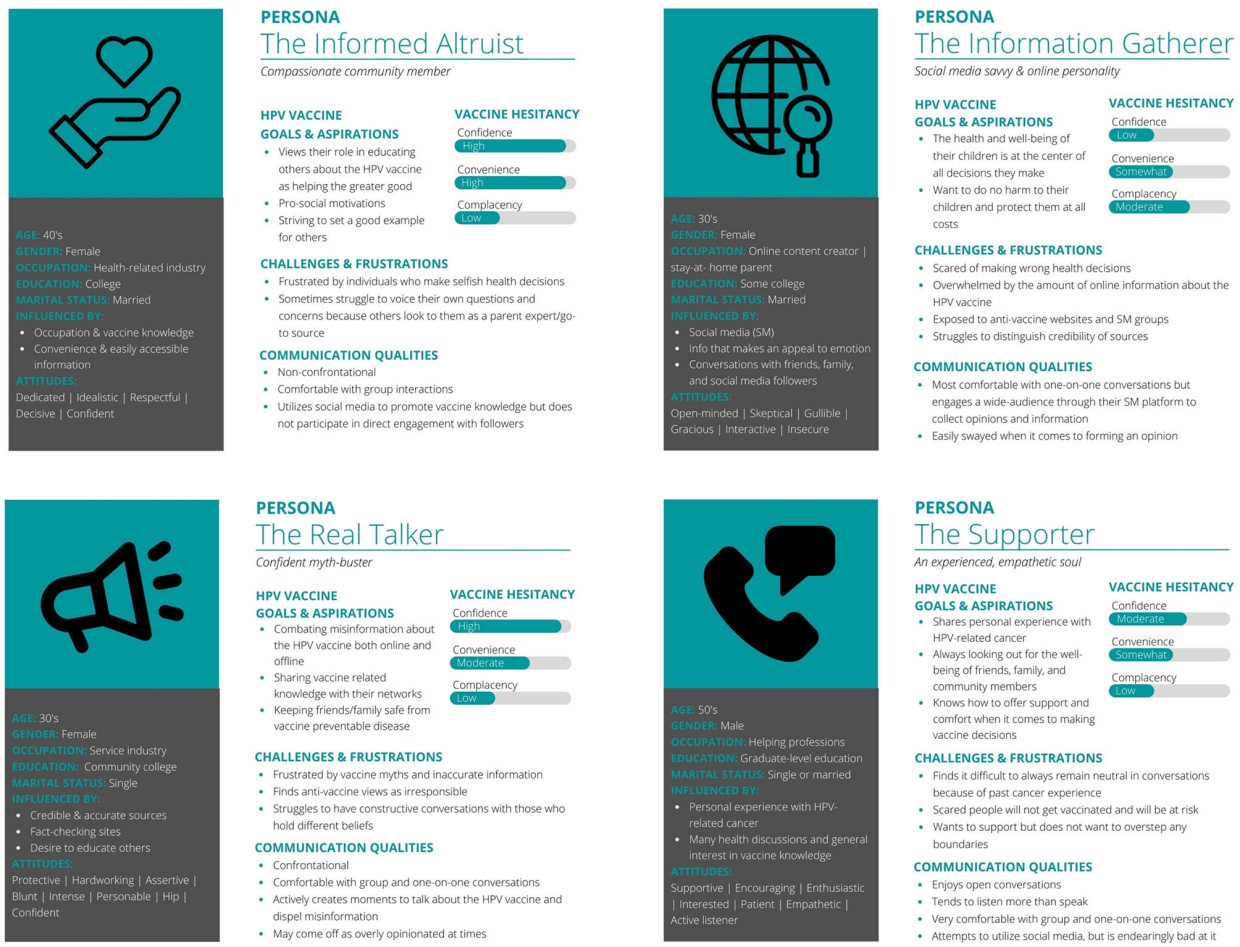
Persona profiles of the informed altruist, the real talker, the information gatherer, the supporter.

Four fictional personas emerged that represent different parent types when it comes to the HPV vaccine and decision making. Displayed by their profiles in Figure 2, the four personas were the (1) Informed Altruist, (2) Real Talker, (3) Information Gatherer, and (4) Supporter.

### The Informed Altruist

The Informed Altruist is a college-educated parent who promotes the collective good of HPV vaccination and has pro-social motivations in all aspects of life. This persona is married, mother to multiple children, and approximately 40 years in age. The informed altruist is a hard-working, managerial-level individual who is kept busy by their work in a health-related field. They prefer an upbeat, fast paced environment. While work is demanding, this persona finds that their spare time is spent getting involved in community groups through their children. If asked to describe their general attitudes and outlook, the informed altruist would say they are compassionate, dedicated, knowledgeable, idealistic, respectful, decisive, and confident. They strive to set positive examples for others, and that is no different when it comes to helping educate parents on the importance of the HPV vaccine, something they care deeply about. Because work and life are time-consuming, this persona greatly values convenience and easy access to credible, reliable information, especially when it comes to health and related topics such as vaccination. Access to reliable information is made easy through their profession and close relationships with others in the field. This makes the informed altruist very knowledgeable about vaccines and confident when it comes to sharing this knowledge with others. Their primary goal regarding the HPV vaccine is to encourage vaccine uptake to benefit the greater good. They enjoy sharing what they know about the HPV vaccine in group settings and take a non-confrontational approach when it comes to discussing differences of opinion regarding the HPV vaccine. The informed altruist also uses their social media platform to educate others but will not directly engage with comments or direct messages. They are most frustrated by individuals who make selfish health decisions and lack understanding of the impact of such decisions. And while this persona is knowledgeable, understanding, and always advocating for the HPV vaccine, they sometimes struggle to voice their own questions and concerns because others look to them as a parent expert and the go-to source. The informed altruist ranked “high” in both confidence and convenience and ranked “low” in complacency.

### The Real Talker

The Real Talker is a curt but personable parent who is driven to inform, educate, and myth-bust when it comes to relaying information about the HPV vaccine to friends and family. This persona is a community college-educated, single mother, in their 30's, who is the primary breadwinner for their family and works in the service industry. They enjoy spending their free time creating memories with friends and family. Despite their primary focus caring for their child(ren), they spend any remaining free time volunteering and getting involved with grassroot activities happening in their community. If asked to describe their general attitudes and outlook, the real talker would say they are protective, hardworking, assertive, blunt, intense, personable, and confident. They are particularly passionate about keeping their family and friends safe and healthy, especially when it comes to preventive health behaviors. They enjoy sharing their informed opinion with others and actively create moments to talk about the health topics they care deeply about—such as the HPV vaccine. Their primary goal regarding the HPV vaccine is to be a strong advocate for vaccination and actively myth-bust and dispel misinformation that they come across in their personal lives and online. They are comfortable having difficult discussions both in group settings and in more intimate one-on-one settings. Because they are blunt and “to the point” they may come off as overly opinionated and, in some cases, railroad conversations that become heated. The real talker is heavily frustrated by online anti-vaccine campaigns and struggle to sympathize and communicate with those who hold anti-vaccine beliefs. Science and fact checking are highly valued and the key evidence this person uses when discussing the HPV vaccine with friends and family. The real talker ranked “high” in confidence, “moderate” in convenience and ranked “low” in complacency.

### The Information Gatherer

The Information Gatherer is an open-minded, yet cautious parent who relies on their online communities and network to learn about the HPV vaccine and help inform their decision making. This persona is a stay at home parent in their late 30's with some college education who has found creative ways to build income through the creation of online content and a social media presence. They enjoy gathering information and opinions online related to various health-focused topics, like the HPV vaccine, and like taking their time when it comes to making health decisions. Because they are the first of their friend group to have children, they are also the first to research recommended vaccinations for children. Through their online community, the information gatherer purposely explores and browses social media outlets that offer varying and opposing opinions to learn about differing perspectives and why parents make certain health decisions. If asked to describe their general attitudes and outlook, the information gatherer would say they are open-minded, gracious, interactive, skeptical, insecure, and flighty. When it comes to determining whether or not the HPV vaccine is necessary for their child, they struggle to sift through the overwhelming amount of information and have a very difficult time identifying what sources to trust. Because of this, they are easily swayed by emotional appeals by anti-vaccine social media campaigns and frequently fall prey to misinformation. This makes it challenging for the information gatherer to provide answers to the questions raised by their child about why they need the vaccine. This persona is also challenged by the overwhelming and conflicting extremes of online health information and will turn to their trusted health care provider for guidance. Ultimately, their primary goal is to make the best decision to protect their child and do no harm to them. The information gatherer ranked “low” in confidence, “somewhat” in convenience and “moderate” in complacency.

### The Supporter

The Supporter is a kind, encouraging, and empathetic parent and grandparent who is known within the community to be the “go-to” person for support as they are well-attuned to the interests and well-being of others. This persona is an older individual in their 50–60's and father to adult children who have multiple of their own. Having had first-hand experience with an HPV-related cancer makes this persona lead with emotion in conversations about vaccine preventable diseases. However, with graduate-level education and having spent the majority of their life in a helping profession, this persona is able to remove the personal element and support others in making their vaccine decisions. If asked to describe their general attitudes and outlook, the supporter would say they are caring, enthusiastic, interested, patient, empathetic, and an active listener. This persona knows how to support others in making vaccine decisions however possible, which is their primary goal. They find joy in having open conversations with family, friends, and community members but tend to listen more than they speak. They are very comfortable having one-on-one conversations about the HPV vaccine and vaccinations in general, though they prefer to have those conversations off-line. The supporter is not very active on social media, but they try to be, and are endearingly bad at it. Overall, this persona finds it hard to remain fully neutral in HPV-vaccine conversations due to past personal experience and hopes to inspire others to get vaccinated so that they are protected and don't have to go through what they did. Their experience with HPV-related cancer makes them a very knowledgeable source and an advocate for trusting the guidance of primary care physicians who want the best for their patients, young and old. The supporter ranked “moderate” in confidence, “somewhat” in convenience and “low” in complacency.

## Discussion

Our findings described the development of four personas that can be used to communicate with parents about the HPV vaccine on social media: the Informed Altruist, the Real Talker, the Information Gatherer, and the Supporter. Developing personas takes data-driven decisions and formative work to identify core values and characteristics that are salient to both the target audience (i.e., parents) and content area (i.e., HPV vaccine). Based on data from a variety of sources, including focus groups, national surveys, and social media, we identified and presented unique beliefs, attitudes, motivations, lifestyles, and communication styles related to the HPV vaccine, synthesized through four personas. While personas have been used in social marketing and health research (32, 41, 42), we found little evidence of persona development for social media interventions.

Personas have been used in a variety of contexts outside of health communication. This approach, describing not only beliefs related to the HPV vaccine but also broader lifestyle behaviors and motivations, highlights the importance of drawing from the fields of marketing and consumer insights (43), where personas have long been used to identify the ideal client or customer (44). Our findings further demonstrate an application of lifestyle marketing on health promotion and communication, by focusing on what people like to do, what they are motivated by, and how they like to spend their free time (45).

Pro-vaccine messaging can be enhanced by using personas to help craft messages and identify narratives for different segments of the population. The personas that we have developed are designed to appeal to parents and are based on data and observations from focus groups, a national survey, social media, and parent input. While this study did not describe message or narrative development, this is the next natural step in the process, using personas to guide and inform ethical message creation (see Figure 1). Each persona will be the foundation for a character, or person, that discusses the HPV vaccine based on the persona experiences and life motivations. Messages will be designed as if they were there being told from each of the characters, making the messages more relatable. The characters will interact with one another and be used to create a story; however, this is not required–personas can also be used to create messages that are standalone that are meant to target people who think or act in a similar way.

The use of social media personas that can provide health information while engaging the target audience is an important tool that can be utilized in public health. By documenting and describing our inputs, process, and outputs of persona development, our process and findings can be applied to other topics in health education and social media research and practice. This strategy can be used not only for health promotion and communication on social media, but also to address the growing and damaging presence of online health misinformation. Many fake social media accounts exist that are designed to accomplish a range of goals, including the spread of health misinformation (46). These accounts can be very successful and often, social media users are unable to identify such accounts (47). The COVID-19 infodemic has shown the need for a research-informed, ethically-based approach to counter inaccurate and biased health information. A model to guide the development of pro-public health personas can offer a way to influence social media users to engage in health promoting behaviors. It is also important to consider how personas can be used to deliver messages that are both culturally competent and health literate to further ensure acceptance and understanding of the information being provided.

Engaging narratives surrounding the personas must also be created to capture the attention of social media users. People are faced with hundreds or even thousands of competing messages every time they engage with their social media feed. The power of narrative in HPV vaccine work has been identified through various studies, including narratives shared through multimedia (16) and interpersonal conversations (17). Extending this work to social media is an important next step and personas are a tool to assist this expansion of the field. In addition, emotional appeal can be heightened through personas and has the potential to address vaccine hesitancy in an important novel way (14). Indeed, research has shown that messages that are emotionally arousing are more likely to be recalled (48), strengthen persuasion (49), and encourage discussion of messages (50). Using images that match the narrative message can also be important for ensuring the message is understood and accepted by the audience (51). Narratives can also help provide social scripts to influence health-related attitudes and behaviors (52).

Our methods are at the intersection of two complex topics: the development of personas to deliver important health information, and vaccination which is known to invoke strong positive and negative sentiment in both traditional and social media. Delving into these multi-faceted topics requires advanced data tools to fully investigate and understand the subject. Mixed methods research, or the integration of both quantitative and qualitative data to answer a single research question (53), is increasingly being seen as a methodology suited for tackling complex public health problems. In our study, we used quantitative data from the National Immunization Survey—Teen to inform us of the primary reasons that parents cited for not vaccinating their adolescent. We collected qualitative data from focus groups and a parent advisory board to better understand those reasons, gather further insight, and explore solutions, all to inform the development of the personas. Additionally, social media data showed us the vaccine messages that parents are exposed to, so that the personas could be responsive to this environment. This robust methodology will ensure that the personas resonate with the parents in our study, as their foundation is quantitative and qualitative data from parents.

Finally, the goal of our project was to develop personas (and as a next step, messages) for parents on Twitter. As such, we focused recruitment of our parent advisory board to parents who were Twitter users and were very familiar with the culture and features of the platform. Future work will want to consider how working with end users from different online platforms may be useful and beneficial to inform project activities on various social media. While these personas are developed specifically for an intervention on Twitter, this approach and actual personas could be (and should be) adapted or applied to various digital environments and social media platforms. Additional personas may also be added for specific parents populations. Furthermore, this approach can be applied to emerging health needs. We have outlined our approach and detailed the ways various data sources have informed the persona development to provide one potential roadmap and methods that can be applied to other health topics with different audiences.

There are a few limitations worth noting. Due to the iterative process of development, not all personas have been ascribed certain demographic characteristics (e.g., race/ethnicity, gender identity/expression, sexual orientation, etc.). This may confer a strength as it will allow for a fluid evolution of the personas to their narrative state in a next phase of message development. Also, personas were developed to communicate information to parents who are still deciding or hesitant about the HPV vaccine and not parents who are completely against the vaccine. In addition, while the personas may capture large segments of the parent population, there are undoubtedly features and characteristics that are not reflected in the four typologies and regular verification can contribute to their expansion and refinement, particularly when applied to different health topics and audiences. Further, though advisory board membership exhibits considerable diversity in race/ethnicity and geography, it may not fully reflect all factors that contribute to parental decisions related to HPV vaccine uptake.

## Conclusions

While other disciplines have used personas for design and marketing, they have been an underutilized tool in health promotion and communication on social media. The ability to capture and communicate both a breadth of experiences and depth of understandings gives this strategy a real potential for impact at both the macro- and micro-levels of influence. Furthermore, combining the use of personas with narratives and storytelling can provide a meaningful and memorable way to organize and communicate health information at the population-level while at the same time relating to individual experiences.

## Data Availability Statement

National Immunization Survey - Teen data is publicly available through U.S. Centers for Disease Control and Prevention at https://www.cdc.gov/vaccines/imz-managers/nis/datasets-teen.html. Other study-related data are available via corresponding author given appropriate ethical approvals are obtained.

## Ethics Statement

The studies involving human participants were reviewed and approved by Drexel University Institutional Review Board. The patients/participants provided their written informed consent to participate in this study.

## Author Contributions

PM, AK, JM, and AL contributed to the conception and design of the study. PM, SC, MRos, ET, and AL contributed to the recruitment of focus groups and parent advisory board. PM, SC, MRoc, ET, AK, JM, and AL performed primary analyses for NIS-teen, social media data, focus group, and parent advisory board workbooks. PM, SC, MRos, RM, and MRoc contributed to the development of personas. All authors contributed to the reporting of the work described in the article and have approved the final version of this manuscript.

## Conflict of Interest

The authors declare that the research was conducted in the absence of any commercial or financial relationships that could be construed as a potential conflict of interest.

## Publisher's Note

All claims expressed in this article are solely those of the authors and do not necessarily represent those of their affiliated organizations, or those of the publisher, the editors and the reviewers. Any product that may be evaluated in this article, or claim that may be made by its manufacturer, is not guaranteed or endorsed by the publisher.
