# Supplementary material for: Development of Personas to Communicate Narrative-Based Information About the HPV Vaccine on Twitter
Source: Front Digit Health. 2021 Aug 4;3:682639. doi: 10.3389/fdgth.2021.682639 (PMC8521793; doi:10.3389/fdgth.2021.682639)
Supplement: Supplementary file 1 [file Data_Sheet_1.PDF]

Project Hashtag HPV

Parent Advisory Board

Materials to Review

★ **Next Meeting: 11/10/2020 from 6:00PM-7:00PM ET**

Thank you for your work on this project! We look forward to getting your feedback on these materials.

Recruitment Twitter Graphics

For the intervention component of this project, we will be recruiting parents from Twitter to participate (similar to how we recruited each of you). We have 8 sample Twitter graphics that we are hoping to get your feedback on (images, wording, etc.).

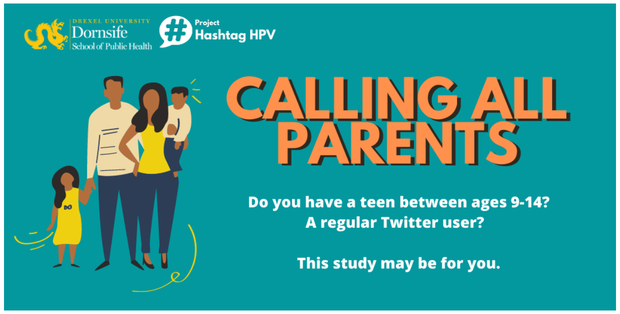

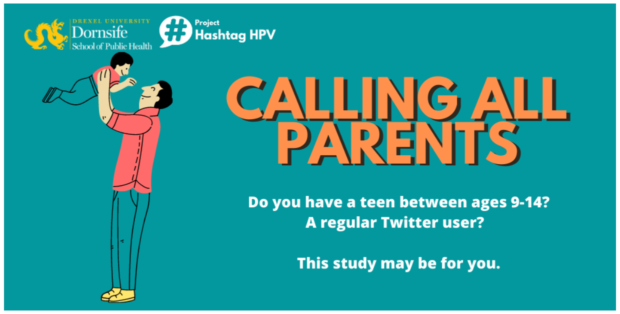

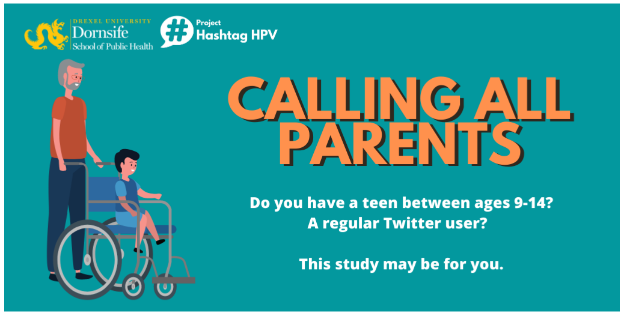

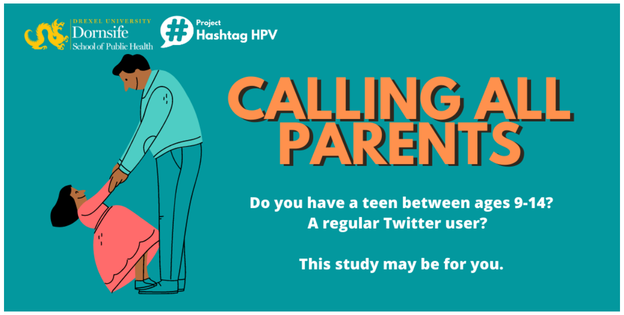

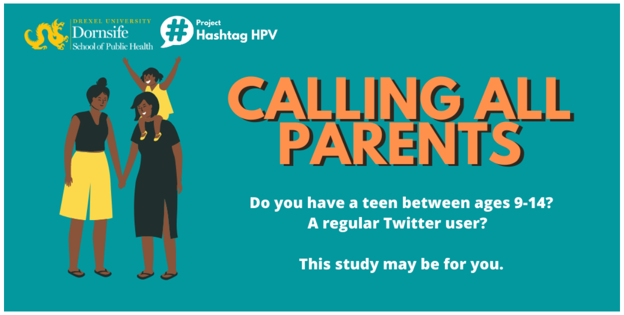

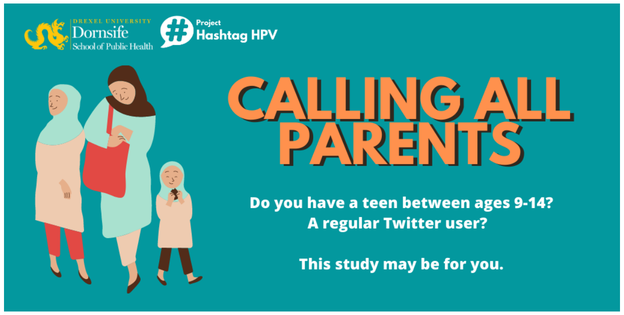

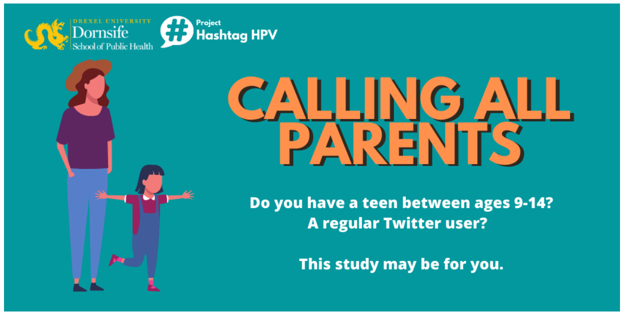

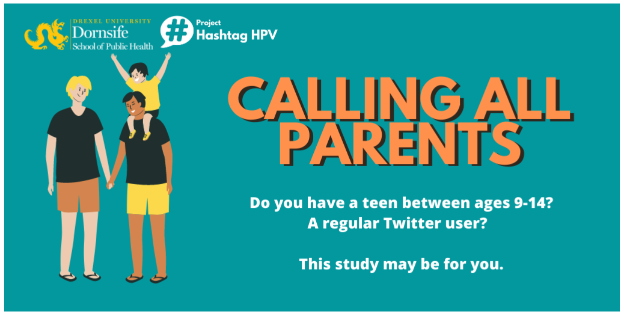

Sample Twitter Ads

Below are two examples of what the ads will look like to parents on Twitter. Please note that the two examples show the same graphic and we will use different graphics when we begin recruitment. The main difference between these two ads is that example 1 includes how much participants will be compensated and example 2 does not.

Example 1

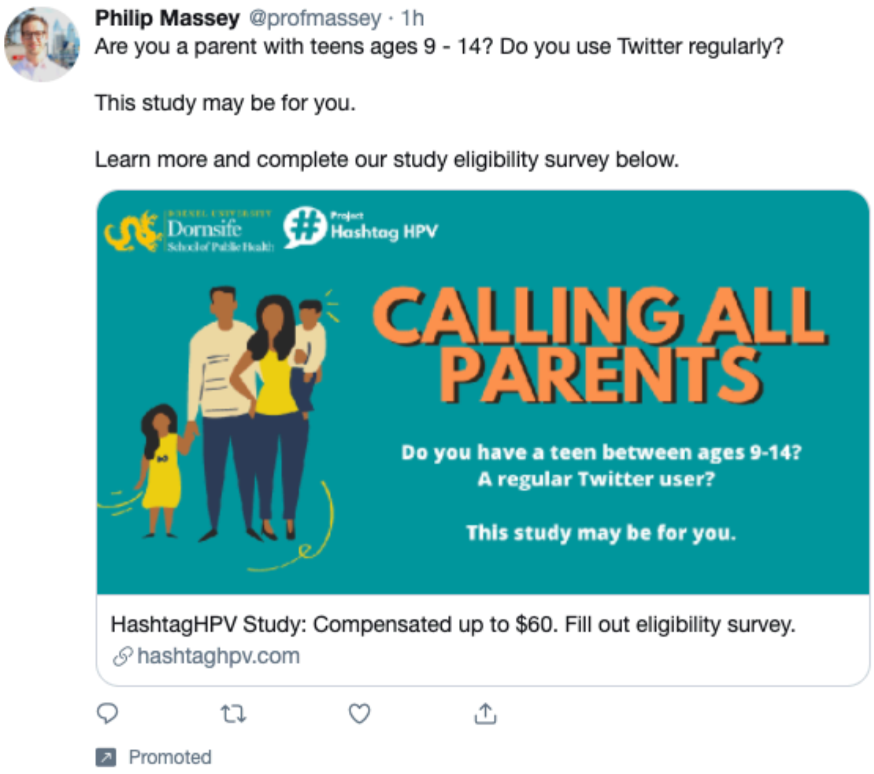

Example 2

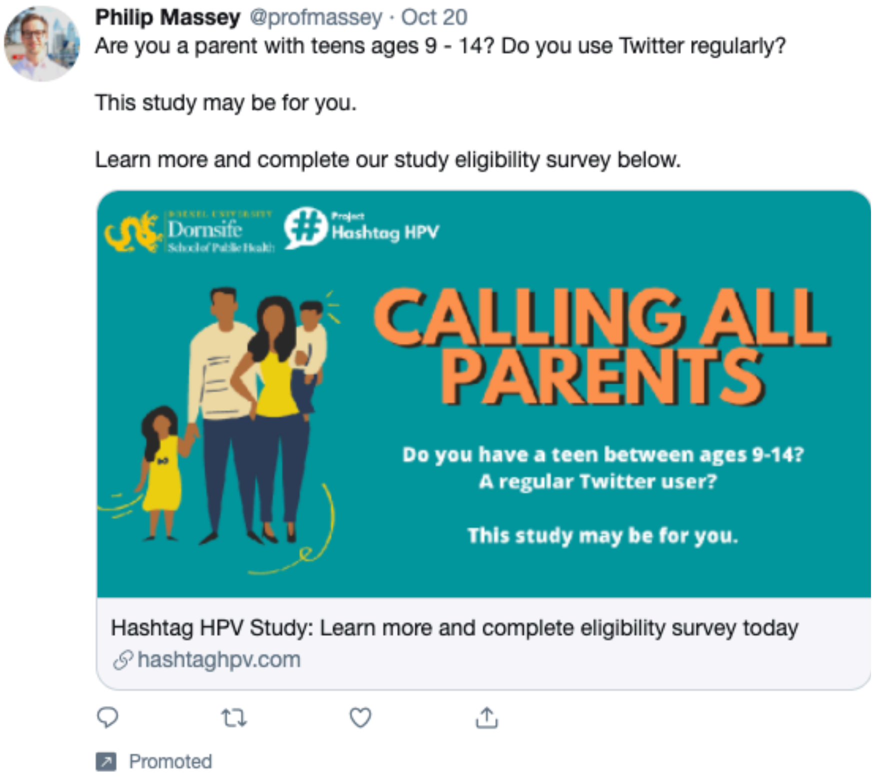

Twitter Ad Feedback

Overall thoughts on the ads:

Do you have any suggestions for language or graphics used in any of the ads?

What do you think of using “Calling All Parents” in the graphics to draw attention? Is there another tagline that you might recommend?

What do you think of the Twitter post examples on the second page? As a reminder, example 1 included the compensation and example 2 did not.

Could you imagine anyone that you know responding to these ads? If you came across this ad, would you respond? Why or why not?

Character Development

The second thing that we would like to get your feedback on is the development of the characters that will "star" in our Twitter messages. As part of the project, participants will either be placed in the "story" group or the "non-story" group. Parents in the story group will read Twitter posts that talk about the HPV vaccine drawing from our character's experience, stories, and evidence. The non-story group, on the other hand, will see posts that focus mostly on evidence, such as numbers and facts, and do not include the character's stories and experiences. The first step in developing the stories is to come up with characters (parents) that the stories will be based on. Each character has its own description. We would like your feedback on each character.

You may be asking – how did you get here? Great question! In the first meeting, we talked about the focus groups that we held with parents in May 2020. The four characters that we have been developing are based on what we learned from the focus groups, and also other data including a national immunization survey. The characters will appear or "star" in the stories that we share on Twitter. The information about the HPV vaccine will be told through their stories and their experiences – we want to give "life" to the information and decisions parents make about the vaccine. This is somewhat similar to when advertisement companies develop commercials for a product, they create characters based on their consumers. This is the same thing that we are doing by creating characters that fit the role of a parent who will need to make a decision about the HPV vaccine for their child.

Below are the four "types" of parents we have started to create. We have not given them names yet, and instead provide you with descriptions. The next page contains a table that further describes the four parent characters that we are developing. After reading through the table about the characters, please provide any feedback that you may have by answering the questions on the page after the table.

We want to incorporate your insights as we continue to develop the four parent character types. We value your opinions and feedback!

The Informed Altruist	The Real-Talker	The Researcher	The Supportive Partner

Categories	The Informed Altruist	The Real-Talker	The Researcher	The Supportive Partner
**Lifestyle**	Involved in many community groups (such as PTA, City Council, etc.). Busy, so convenience is important to them.	Works very hard to support family; spends free time with friends and family. Has been involved in grassroots activities.	Enjoys gathering information and taking their time to make a decision. Scared of making the wrong decision. May be asked by others to share their info.	Has a job that keeps them pretty busy; not a lot of time to research vaccines
**Characteristics**	Compassionate, dedicated, analytical, idealistic, altruistic	Protective, hardworking, assertive, blunt	Open-minded, uncertain, gullible, gracious, trusting	Busy, supportive, encouraging, passive
**Motivations**	Helping the greater good	Keeping their family/friends safe and healthy; Myth-busting	Make the best decision to protect their child and do no harm to them	Supporting and reassuring their partner; keeping their child healthy and safe
**Triggers, what upsets them**	Selfish health decisions	Spreading misinformation/vaccine myths; not thinking for yourself	Conflicting extremes of information; lack of strong doctor recommendation	Does not want to take the lead on making any health decisions for their child
**Confrontation/ interaction**	More non-confrontational, more comfortable with broad conversations/ announcements	Comfortable with one-on-one confrontation. Willing to engage in difficult conversations to support "shared learning"	More non-confrontation due to lack of confidence, but very interactive.<br>Asks others/friends about their vaccine opinions and actions	Relies on communication with partner and If partner has doubts, they have doubts.
**Actions**	Dedicated to informing as many people as they can about vaccinating	Will inform the many, but will inform/debate with others at the individual level	Will discuss information they come across, using friends and family as a sounding board	Passive; partner does most research about health decisions
**Vaccine knowledge**	Knows a lot about vaccine efficacy, necessity, and safety. Very confident in their knowledge.	Is generally knowledgeable about vaccine efficacy, safety, and side effects.	New to vaccine information, and may find it challenging to distinguish good and bad sources	Not very knowledgeable, may not understand why vaccine is recommended for 11-12-year-old – seems young.
**Social media**	Uses social media to promote importance of vaccines, but may not engage directly with others	Frustrated that vaccine misinformation is widespread on social – will engage directly to inform others and dispel myths	May come across and/or believe anti-vaxx information while researching on social media. Overwhelmed and at times confused at the misinformation and controversy around vaccination.	May come across information passively on social media, may share content with partner.

Character Feedback

Specific thoughts on "The Informed Altruist"

Specific thoughts on "The Supportive Partner"

Specific thoughts on "The Researcher"

Specific thoughts on "The Real-Talker"

These characters are still in the development phase. What would you add or change about the descriptions of the characters? Are there any other categories that you might add?

Would parents that you know listen to any of these characters? Why or why not?

Would parents that you know believe any of these characters if they were talking to them about vaccines? Why or why not?

Have you ever known anyone like any of these characters? Would you identify with any of these characters?

Please share any other thoughts or feedback you may have here on the Twitter ads and the characters.
