# Supplementary material for: Development of Personas to Communicate Narrative-Based Information About the HPV Vaccine on Twitter
Source: Front Digit Health. 2021 Aug 4;3:682639. doi: 10.3389/fdgth.2021.682639 (PMC8521793; doi:10.3389/fdgth.2021.682639)
Supplement: Supplementary file 2 [file Data_Sheet_2.PDF]

Project Hashtag HPV

Parent Advisory Board

Materials to Review

Next Meeting: 12/15/2020 from 6:00PM-7:00PM ET

Thank you for your work on this project! We look forward to getting your feedback on these materials.

Recap

In the last meeting, the two main project components that we focused on were the recruitment Twitter ads and the characters. We refined the ads based on your feedback and below you will see a couple of examples. For this workbook, we are asking for your feedback on the characters and will plan to spend the majority of the upcoming meeting refining their descriptions.

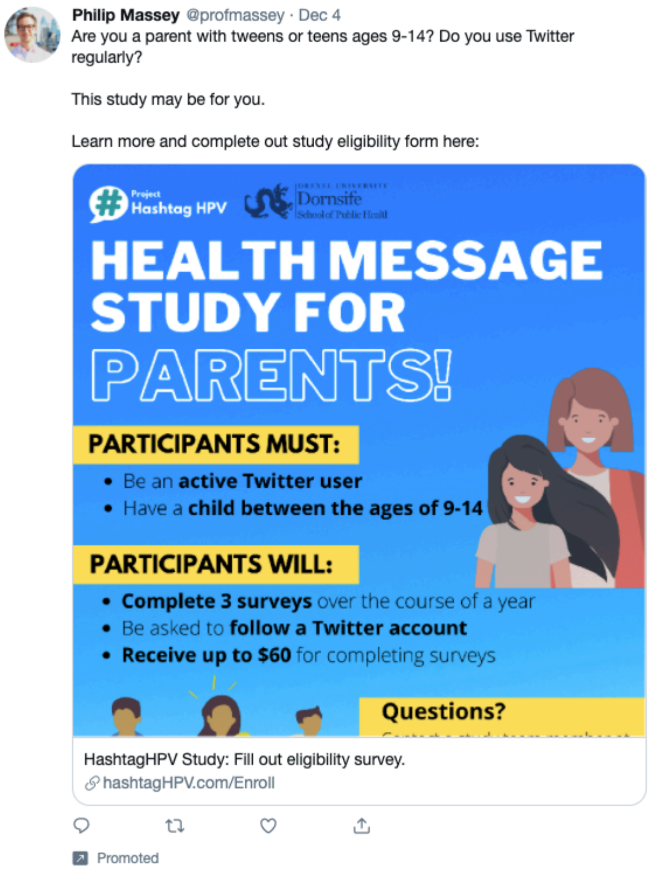

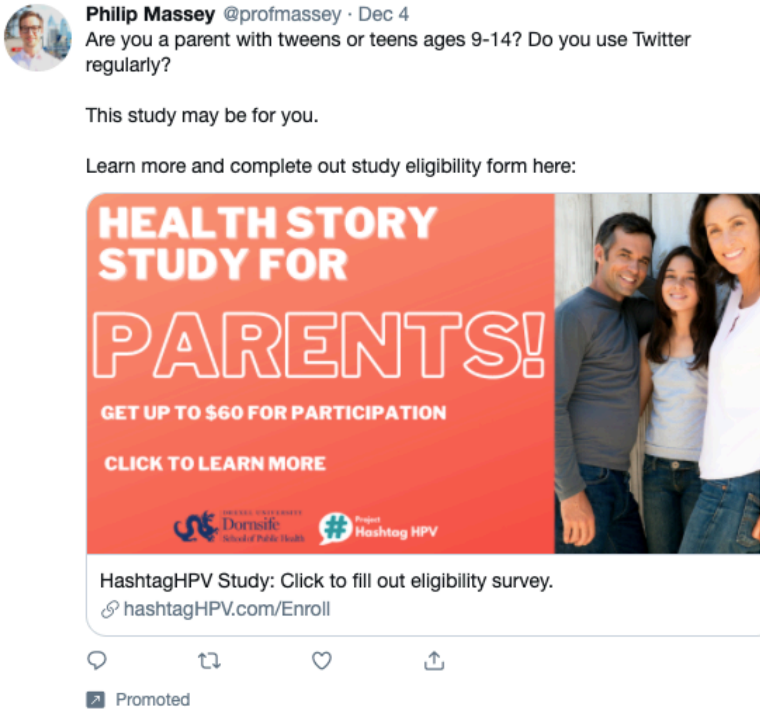

Character Development

The main focus of this workbook and meeting 3 is the characters. You provided us great feedback and we were able to make a lot of changes. We are hoping to get your thoughts on the changes and the overall descriptions.

As a reminder, the research study that we are testing is a Twitter intervention that focuses on the HPV vaccine for parents. We plan to enroll 600 parents who will be divided into 2 groups, the **story group** and the **non-story group**. Parents in the story group will read Twitter posts that talk about the HPV vaccine through stories based on the characters that we are developing. The information about the HPV vaccine will be told through their stories and their experiences. We want to give "life" to the information and decisions that parents make about the vaccine. The non-story group will see posts that focus mostly on evidence, such as numbers and facts, and do not include the character's stories and experiences.

The following pages contain the revised descriptions of the 4 characters with questions for you to answer after each description.

The Informed Altruist	The Real-Talker
The Information Gatherer	The Supportive Parent

The Informed Altruist

Characteristics

- Altruistic, compassionate, dedicated, knowledgeable, idealistic, respectful.
- Non-confrontational: Comfortable with broad conversations/announcements (e.g. to groups instead of individuals).

Description/ Lifestyle

- Works in the medical field.
- Involved in many community groups (PTA, City Council, etc.).
- Often overcommits themselves and takes on more than they can handle because they're interested/passionate about many things.
- They need quick/readily accessible information due to busy lifestyle.
- Motivated by social altruism.
- Dedicated to informing as many people as they can about vaccinating to promote herd immunity.
- Believes vaccinating is a civic duty.
- Upset by anti-vaxx websites and health care decisions that prioritize self over others.

Contextual Influences

- Gets information about vaccine from reputable sources, such as CDC, other local government agencies, their primary care provider, colleagues, etc.
- Inconvenient appointment times for vaccinating may dampen motivation (e.g. during school hours).
- Sometimes it is hard for them to make and attend appointments due to busy schedule, and this can be even more difficult as appointments may change (due to COVID for instance).

Individual/ Group Influences

- Believes that vaccinating is to promote public health and well being of others (motivated by greater good vs. self).
- Very knowledgeable about vaccine efficacy, necessity, and safety.

Key Struggle/ Challenges

- Despite knowledge and strong belief in vaccine, may have hesitation at moment of vaccine administration and may not know how to address this "unanticipated" emotional response.
- May feel pressure from the field or their "circle of friends" and hardly ever thinks twice about any vaccine related decision. They may realize that they do have more feelings about vaccines (imposter syndrome).
- Child may bring up questions about the vaccine and parent may want to give more autonomy and allow their child to work through their questions.

The Informed Altruist Feedback

Who in your life reminds you of The Informed Altruist (you or someone else)? What are some defining characteristics of this person (fears, struggles, strengths, relationships with other people, etc.)?

Do you know if the person that you are thinking of has gotten their child the HPV vaccine? How did you find out? Do you know anything about their decision-making process or experience with getting the vaccine?

What are some of this persons greatest strengths (think about their characteristics)? What are some things that they struggle with?

When you read the description is there an image that you have in mind for The Informed Altruist? Describe how you picture this person.

The Real-Talker

Characteristics

- Protective, hardworking, assertive, blunt, personable, hip, confident.
- Comfortable with one-on-one discussion and engaging people in difficult conversations (especially about vaccines).

Description/ Lifestyle

- Primary breadwinner for family.
- Spends free time with friends, co-workers, and family.
- Has been and is involved in grassroots activities.
- Wants to vaccinate to ensure the health and safety of family and friends.
- Frustrated that vaccine myths and misinformation are so widespread.
    - Actively tries to dispel them if they are brought up in person or online.
    - Views anti-vaxxers as ridiculous and does not understand how they could be so easily manipulated.
    - Wants to speak out against anti-vaxx misinformation before people are influenced by it.
- Values independent thinking.

Contextual Influences

- Wary of judgmental PCP/vaccination situations.
- Possible lack of transportation/cost of traveling to the clinic.
    - Convenience in accessing the vaccine is important to them.

Individual/ Group Influences

- Is generally knowledgeable about vaccine efficacy, safety, and side effects.
- Believes in necessity and efficacy of vaccines, but fact-checks all information (PCP decisions, etc.)
- Enjoys the back-and-forth of discussions that can lead to learning and understanding – will learn from others and is also happy to help others learn.
- Appreciates when people they are talking to are independent thinkers; when others want more information, they find it empowering.
- Anti-vaxx websites/social media push them further in support of vaccinating

Key Struggle/ Challenges

- They may struggle to talk to others who have differing/strong opinions about vaccines. They are very confident in the information that they have researched and may not be as understanding when others have opposite opinions.
- Child may bring up questions about the vaccine and they do not want them to think that getting the vaccine is optional. They may be more assertive about vaccines, which could result in their child not feeling heard.

The Real-Talker Feedback

Who in your life reminds you of The Real-Talker (you or someone else)? What are some defining characteristics of this person (fears, struggles, strengths, relationships with other people, etc.)?

Do you know if the person that you are thinking of has gotten their child the HPV vaccine? How did you find out? Do you know anything about their decision-making process or experience with getting the vaccine?

What are some of this persons greatest strengths (think about their characteristics)? What are some things that they struggle with?

When you read the description is there an image that you have in mind for The Real-Talker? Describe how you picture this person.

The Information Gatherer

Characteristics

- Open-minded, uncertain, gullible, gracious, trusting, interactive.
- Open to having difficult conversations, but may become overwhelmed with information.

Description/ Lifestyle

- Spends a lot of time becoming knowledgeable (e.g. reading articles and watching videos on social media).
- Large and wide social network, often at center.
- More hands on in kids' lives, may be involved in many or all aspects from school to sports to clubs to service (e.g. member of PTA or class parent).
- Spends a lot of time thinking about parenting and kids.
- Enjoys gathering information and taking their time to make decisions.
- Spends a lot of time advocating for children.
- The first member of their friend group to research/consider vaccination; trailblazer.
    - Uses friends and family as a sounding board for new information they encounter.
    - Frequently asks other people about their vaccine options and actions.
    - Friends and family may ask the Researcher to share information they've found.
- Scared of making the wrong decision due to this being their first time researching the vaccine.

Contextual Influences

- Ready and wanting a provider recommendation to vaccinate.
- Sees provider as another data point or source for helping them sift through information.
- If transportation or cost is a barrier, they may not think it's worth their rime/energy to get their child vaccinated.
- Influenced by celebrities and popular culture.

Individual/ Group Influences

- Lack of knowledge about how vaccines work,
- May not have scientific/academic background and know how to distinguish good and bad sources.
- Decision to vaccinate is more about their kids than the greater good.
- Anti-vaxx websites/Facebook groups may plant seeds of doubt about safety, efficacy, and role of pharmaceutical [illegible]

Key Struggle/ Challenges

- Tends to become overwhelmed with conflicting information and may have a hard time making decisions about vaccination for their children.
- When their child asks them questions about vaccines, they have a hard time responding because they do not have the answers themselves. This sometimes leaves their child confused or feeling less confident.

The Information Gatherer Feedback

Who in your life reminds you of The Information Gatherer? What are some defining characteristics of this person (fears, struggles, strengths, relationships with other people, etc.)?

Do you know if the person that you are thinking of has gotten their child the HPV vaccine? How did you find out? Do you know anything about their decision-making process or experience with getting the vaccine?

What are some of this persons greatest strengths (think about their characteristics)? What are some things that they struggle with?

When you read the description is there an image that you have in mind for The Information Gatherer? Describe how you picture this person.

The Supportive Parent

Characteristics

- Supportive, encouraging, enthusiastic, interested, patient, empathetic, active listener.
- Enjoys having open discussions with people and tends to do most of the listening in conversations.

Description/ Lifestyle

- Could provide emotional support and guidance for parents (similar to a therapist).
- Could be a neighbor, cousin, aunt/uncle, grandparent, friend, etc.
- Supports parents researching vaccines as much as possible. Will do research with other parents if asked for a second opinion. Is also willing to share their personal experiences.
- If partner or other parent they are supporting has doubts, they will help them navigate the doubts.
- Understands that some decisions can be hard, but also feels confident in supporting the right decision.
- Is interested in the lives and wellbeing of others and tries to support them (by checking in to see how they are doing, watching people's kids when they can't get a babysitter, etc.).

Contextual Influences

- Encourages parents to trust their PCP for big health decisions like vaccination for their child/children.
- Spends a lot of free time helping other parents.

Individual/ Group Influences

- Comfortable with interpersonal communication, talking to others one-on-one.
- May come across information passively on social media, and may share with other parents if they seem interested.

Key Struggle/ Challenges

- Has a hard time saying no to people. Sometimes overcommits to people and then forgets to take care of themselves.
- Tries to remain neutral when talking to other parents about vaccines, but can sometimes be difficult due to personal experience.
- Child may bring up questions about the vaccine and they want to support their child's interest and may look up information together on the vaccine. Asks how child is feeling about getting the vaccine.

The Supportive Parent Feedback

Who in your life reminds you of The Supportine Parent (you or someone else)? What are some defining characteristics of this person (fears, struggles, strengths, relationships with other people, etc.)?

Do you know if the person that you are thinking of has gotten their child the HPV vaccine? How did you find out? Do you know anything about their decision-making process or experience with getting the vaccine?

What are some of this persons greatest strengths (think about their characteristics)? What are some things that they struggle with?

When you read the description is there an image that you have in mind for The Supportive Parent? Describe how you picture this person.

Overall Feedback

**Eventually, we will create visual representations of these characters. When we do create the images, would you prefer the person to be a real person, a cartoon avatar, or something else?**
**Out of all 4 characters, is there one that you could see being the main character in the storyline? Why or why not?**
**Overall, is there anything that you would add or change to any of the characters?**
**Please share any other thoughts or feedback that you have here.**
